# General Practitioners and Hospital Residents

**Published:** 1907-05-04

**Authors:** 


### Resident Medical Officers' Department.

### GENERAL PRACTITIONERS AND HOSPITAL RESIDENTS.

We can only deal in this article with one or two aspects of the relations between general practitioners and hospital residents. As we have already said, in the introduction to this section of The Hospital, whenever a dispute arises between a house surgeon and a general practitioner, and the matter is taken up by the Press, the house surgeon seldom has full justice done to him. This is because in the particular case in point he is generally in the wrong. But if the circumstances of many preceding cases, which have not been made public, were known, it would generally be found that there was another side to the question. We will try to show what this other side is. To those acquainted with the inner workings of hospital life we have nothing to tell that they do not already know. We merely wish to state without prejudice the case for the house surgeon.

The dispute commonly arises out of a case which a general practitioner has sent up to be admitted into hospital, and which the medical officer on duty, although having beds at his disposal, does not consider suitable for admission, and therefore refuses to admit. If the house surgeon is correct in his opinion of the case, the matter usually drops, except for a feeling of resentment on both sides. If the doctor is right, and the case is really suitable for immediate admission, and if, further, the patient comes to harm on account of the house surgeon's refusal, the matter probably becomes public. Perhaps the patient dies, and a certificate is withheld, or the coroner or the police are informed of the circumstances. The proceedings at the inquest reveal the fact that a junior practitioner, the servant of a public institution, has set his opinion against that of a colleague of far greater experience, and has, by so doing, at least hastened a patient's death. All this is perhaps quite true. The house surgeon is censured by the coroner's jury and by his own hospital authorities; the medical Press raises up its voice in protest against such presumptuous conduct; and the lay papers make helpful suggestions for the better management of our hospitals, including perhaps a recommendation that no authority should be entrusted to medical officers under a certain age. After a while the subject is dropped, and all that remains in the public mind is a vague impression that things are not quite as they should be with the hospitals.

Now for the other side of the question: the side known only to those who are or have been behind the scenes of hospital life. It is a frequent experience for the house surgeon on duty, with only one or two vacant beds at his disposal, and these marked for the reception of any urgent cases which may arrive during his period of taking in, to be told by the night porter that a case sent up by an outside doctor has arrived in an ambulance. With the ambulance attendant is a note to the effect that the patient is urgently ill, and is in the doctor's opinion suitable for instant admission. This is perhaps the only information supplied; perhaps there is the vague but suggestive diagnosis of an "acute abdomen," an expression only current during the past year or so, and one which often covers a multitude of professional sins. In extreme cases, these notes are masterpieces of reticence. Time after time these so-called urgent cases have been admitted into the precious beds reserved for emergencies, only to turn out on examination to be trivial, or chronic, or otherwise unsuitable. However, the patient is at the door, and without careful investigation, the urgency or otherwise of his condition cannot be made out. The house surgeon, with some misgivings, orders the case to be brought into the receiving room. He cannot make out that there is much the matter, but he remembers that the exposure and excitement of travelling from bed to hospital often obscure important symptoms. The hour is late and his colleagues are asleep. Once inside the hospital and labelled by an outside practitioner as urgently ill, the patient is already as good as admitted. The ambulance driver wishes to get back to his bed—he may have miles to go to return to his stables in the suburbs—and he asks if it is necessary for him to stay any longer. The house surgeon hesitates and is lost. The patient is admitted, and shortly afterwards a really urgent case arrives and has to be sent elsewhere: and next day the house surgeon is the laughing-stock of the hospital for having admitted into the only vacant surgical bed a case, perhaps, of chronic constipation with the diagnosis of intestinal obstruction, or a pregnant woman as a case of appendix abscess.

The above illustration may seem very much overdrawn; nevertheless it is a faithful composite picture of many actual instances which have occurred in the writer's own experience. It is fairly typical of the cases sent up to hospital in ambulances by certain doctors practising in or around great cities. Some of these practitioners are well known to hospital residents for palming off cases of this sort upon unwary house surgeons; not with intent to deceive, let us hope, but because they neither know what is the matter with their patients nor what to do with them, and the hospital wards offer the easiest solution of the difficulty. It is small wonder that an overworked suburban practitioner sometimes makes use of this expedient in the case of an exacting patient, who is never likely to pay a fee, and whose friends are only too willing to get rid of the trouble of looking after him. But it is not fair to make a practice of bouncing such cases into hospitals. Thus it is that cases sent up by certain outside doctors are sometimes wrongly refused admission. In one hospital a "black list" has even been drawn up by the residents, and one can hardly blame them for fighting shy of any patient who is sent up for instant admission by a doctor whose name is inscribed on this roll of fame. In some hospitals, again, the telephone is a constant source of annoyance and perplexity to the junior staff. The same practitioners who send up unsuitable cases in cabs or ambulances, weary of finding these patients returned to them without thanks, resort to the ingenious plan of ringing up the house surgeon on duty at one hospital after another, until someone, fresher or weaker than the rest, is convinced by their eloquence, and promises a bed for the patient. This is what is known as the "telephone curse," and may explain why it is almost impossible for general practitioners nowadays to get into direct communication with a hospital resident who is not known to them by name. We have said enough to show that there is some excuse for the resident medical officer when he occasionally makes a mistake which brings the Press and the public about his ears for refusing admission to a genuinely urgent case which has been sent to him by a general practitioner. Having been fifty times bitten, he is rather to be pitied than to be blamed if he comes to grief on the one occasion on which he turns shy. It is much to be regretted that, owing to the methods adopted by a few members of their own class, the general practitioners in certain districts should regard hospital residents as their natural enemies, and that for the same reason some of our hospital residents should return the compliment.

